# Reducing brain TACE activity improves neuroinflammation and cardiac function in heart failure rats

**DOI:** 10.3389/fphys.2022.1052304

**Published:** 2022-11-09

**Authors:** Yang Yu, Baojian Xue, Nafis Md Irfan, Terry Beltz, Robert M Weiss, Alan Kim Johnson, Robert B Felder, Shun-Guang Wei

**Affiliations:** ^1^ Department of Internal Medicine, University of Iowa, Iowa City, IA, United States; ^2^ Psychological and Brain Sciences, University of Iowa, Iowa City, IA, United States; ^3^ Abboud Cardiovascular Research Center, University of Iowa, Iowa City, IA, United States; ^4^ Iowa Neuroscience Institute, University of Iowa Carver College of Medicine, Iowa City, IA, United States; ^5^ VA Medical Center, Iowa City, IA, United States

**Keywords:** hypothalamic paraventricular nucleus, sympathetic nervous system, inflammation, ADAM17, tumor necrosis factor-α, myocardial infarction

## Abstract

Tumor necrosis factor (TNF)-α converting enzyme (TACE) is a key metalloprotease mediating ectodomain shedding of a variety of inflammatory mediators, substrates, and growth factors. We previously reported that TACE-mediated production of TNF-α in the hypothalamic paraventricular nucleus (PVN) contributes to sympathetic excitation in heart failure (HF). Here, we sought to determine whether central interventions in TACE activity attenuate neuroinflammation and improve cardiac function in heart failure. Myocardial infarction-induced HF or sham-operated (SHAM) rats were treated with bilateral paraventricular nucleus microinjection of a TACE siRNA or a 4-week intracerebroventricular (ICV) infusion of the TACE inhibitor TAPI-0. Compared with SHAM rats, scrambled siRNA-treated HF rats had higher TACE levels in the PVN along with increased mRNA levels of TNF-α, TNF-α receptor 1 and cyclooxygenase-2. The protein levels of TNF-α in cerebrospinal fluid and phosphorylated (p-) NF-κB p65 and extracellular signal-regulated protein kinase (ERK)1/2 in the PVN were also elevated in HF rats treated with scrambled siRNA. The expression of these inflammatory mediators and signaling molecules in the PVN of HF rats were significantly attenuated by TACE siRNA. Interestingly, the mRNA level of TNF-α receptor 2 in the PVN was increased in HF treated with TACE siRNA. Moreover, sympathetic excitation, left ventricular end-diastolic pressure, pulmonary congestion, and cardiac hypertrophy and fibrosis were reduced by PVN microinjection of TACE siRNA. A 4-week treatment with intracerebroventricular TAPI-0 had similar effects to ameliorate these variables in HF rats. These data indicate that interventions suppressing TACE activity in the brain mitigate neuroinflammation, sympathetic activation and cardiac dysfunction in HF rats.

## Introduction

Increased pro-inflammatory cytokines (PICs) in the periphery and the brain have been associated with an adverse prognosis in systolic heart failure (HF) (Dibbs et al., 1999; Deswal et al., 2001; Yu et al., 2019b; Diaz et al., 2020; Badoer, 2022). Tumor necrosis factor-α (TNF-α), one of the key PICs with pleiotropic effects, plays an important role in the pathogenesis of HF. In patients with HF, increased circulating levels of TNF-α are closely correlated with the severity of HF and are sufficient to cause cardiac dysfunction (Francis, 1999; Hori and Yamaguchi, 2013). Additionally, increased levels of TNF-α in the brain have been shown to induce neuroinflammation, which leads to sympathetic activation, an important contributor to the progression in animal model of systolic HF (Kang et al., 2009; Wei et al., 2016; Yu et al., 2019a; Yu et al., 2022a; Badoer, 2022). Interventions reducing the levels of TNF-α or its receptor in the brain can effectively attenuate sympathetic excitation and ameliorate peripheral manifestations of HF (Guggilam et al., 2011; Yu et al., 2017). However, clinical trials using the TNF-α inhibitors etanercept or infliximab have failed to demonstrate clinical benefits (Anker and Coats, 2002; Chung et al., 2003). These unsuccessful clinical trials call attention to the need for a better understanding of the inflammatory mechanisms driven by TNF-α in HF, so that alternative therapeutic approaches can be developed to counter its adverse effects.

The proinflammatory soluble form of TNF-α (sTNF-α) is produced from its precursor, transmembrane-TNF-α (tmTNF-α), *via* a process called ectodomain shedding mediated by TNF-α converting enzyme (TACE, also known as ADAM17) (Kriegler et al., 1988; Moss et al., 1997). tmTNF-α is proteolytically cleaved by TACE at the cell surface to release sTNF-α (Black et al., 1997). The ectodomain shedding of sTNF-α by TACE is critical for TNF-α–induced inflammation in pathological states and inflammatory diseases including HF and hypertension. Elevated expression of TACE in the heart and circulation is associated with cardiac remodeling in rodent models of myocardial infarction (Zheng et al., 2016; Hasan et al., 2020). In patients with acute myocardial infarction, TACE in the circulation is increased and correlates with infarction size and the severity of HF (Akatsu et al., 2003; Shimoda et al., 2005). The elevated myocardial sTNF-α is accompanied with increased TACE levels in the circulation and in myocytes, suggesting that TACE is an important mediator for TNF-α production in the development of human HF. While sTNF-α exerts proinflammatory action, tmTNF-α has been reported to play an anti-inflammatory role to counter the adverse effects of TNF-α (Canault et al., 2004; Olleros et al., 2012; Green et al., 2016). Targeting TACE to reduce sTNF-α production while sparing tmTNF-a has potential therapeutic importance in treating HF in the clinical setting.

Over the past decade, the role of neuroinflammation as driver of autonomic nervous system activation and pathological neurohumoral responses in the progression of HF has been increasingly appreciated (Wei et al., 2012; Diaz et al., 2020; Badoer, 2022). We previously demonstrated that TACE is abundantly expressed in the brain and upregulated in the hypothalamic paraventricular nucleus (PVN) and the subfornical organ in rats after myocardial infarction (Yu et al., 2019a). Central blockade of TACE activity in the PVN reduced hemodynamic responses and sympathetic excitation in rats with HF (Yu et al., 2019a). Upregulation of TACE in HF may account for the increased sTNF-α, and possibly other inflammatory mediators *via* the cytokine cascade, in these autonomic/cardiovascular brain regions, and may contribute importantly to neurohumoral excitation in HF rats. Notably, tumor necrosis factor receptor 1 (TNFR1), the main receptor mediating the inflammatory effects of sTNF-α, is also upregulated in the brain in HF (Yu et al., 2017). Central injection of TACE in HF rats induces substantial increases in hemodynamic responses and sympathetic outflow, which can be prevented by a TACE inhibitor or a TNF-α inhibitor (Yu et al., 2019a).

In the present study, we sought to determine whether central blockade of TACE by genetic knockdown of TACE with a TACE siRNA in the PVN, or by pharmacological inhibition of TACE activity with its inhibitor, attenuates TNF-α-activated signaling pathways to reduce neuroinflammation and sympathetic excitation in HF animals. We further examined the effects of these central manipulation of TACE on cardiac dysfunction and remodeling during the progression of HF.

## Materials and methods

### Animals

Adult male Sprague-Dawley rats (250–300g) were obtained from Envigo/Harlan (Indianapolis, IN). Animals were housed in a temperature- (23 ± 2°C) and light-controlled animal care facility, and standard rat chow and water were given *ad libitum*. Experiments were performed in accordance with the National Institutes of Health Guide for the Care and Use of Laboratory Animals. All procedures (protocol #: 0062031) were approved by the Institutional Animal Care and Use Committee of the University of Iowa. All efforts were made to minimize the number of animals used and their suffering.

## Experimental protocols

### Protocol I: Genetic knockdown of tumor necrosis factor-α converting enzyme in the paraventricular nucleus by tumor necrosis factor-α converting enzyme siRNA AAV virus

Rats anesthetized with ketamine plus xylazine (100 + 10 mg/kg) underwent bilateral PVN microinjections of an adeno-associated virus 9 (AAV9) vector encoding either a TACE siRNA (0.15 µl of 10^13^ GC/ml per side, *n* = 48) or a scrambled (Scr) siRNA (n = 46), and green fluorescent protein (GFP) as previously described (Yu et al., 2022a). One week later, some rats that had received PVN microinjection of AAV-TACE siRNA (n = 15) or AAV-Scr siRNA (n = 12) were euthanized by decapitation to collect brain to verify the transfection potential and the knockdown efficiency of AAV-TACE siRNA. Twelve untreated age-matched rats served as controls (CON).

Under ketamine plus xylazine (100 + 10 mg/kg) anesthesia, the remaining animals that had received AAV-TACE siRNA or AAV-Scr siRNA underwent coronary artery ligation (CL) to induce HF or a sham operation (SHAM). Left ventricular (LV) function and infarction size were assessed by echocardiography within 24 h of CL or SHAM. HF rats were evenly assigned to the treatment group or control group based on the infarction size to assure that comparisons across groups reflect HF of similar severity. 3 rats with a small ischemic zone (≤20%) were excluded from further study. 11 HF rats died before the end of the experiments. The surviving HF and SHAM animals were assigned to four treatment groups: 1). HF + AAV-TACE siRNA (n = 13); 2). HF + AAV-Scr siRNA (n = 14); 3). SHAM + AAV-TACE siRNA (n = 13); and 4). SHAM + AAV-Scr siRNA (n = 13).

**FIGURE 1:**
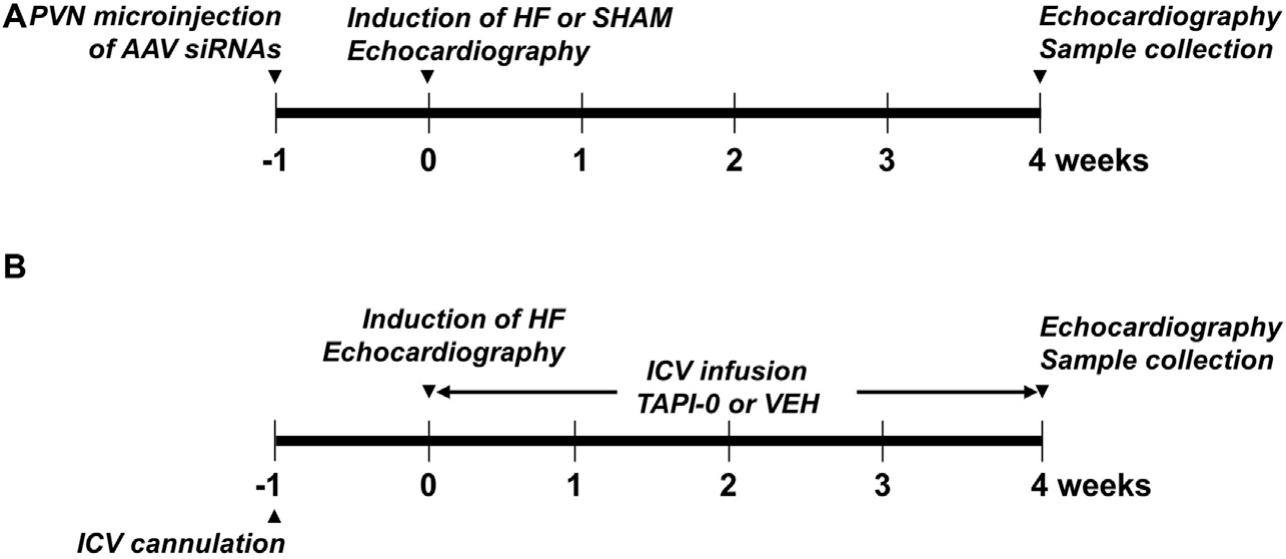
Schematic showing the experimental procedures in protocol I **(A)** and Protocol II **(B)**.

After a second echocardiogram was performed to determine treatment effects at 4 weeks, these rats were anesthetized with urethane (1.5 g/kg, ip) for assessments of cardiac hemodynamics and collection of cerebrospinal fluid (CSF), and then euthanized by decapitation to harvest brain and blood for molecular studies. The heart and lungs were also collected and weighed to assess peripheral indicators of HF. The experimental procedures in this protocol are summarized in Figure 1A.

### Protocol II: Central blockade of tumor necrosis factor-α converting enzyme activity by its inhibitor TAPI-0

Rats were implanted with stainless steel cannulas in the lateral cerebral ventricle under anesthetized condition by ketamine plus xylazine (100 + 10 mg/kg) as previously described (Wei et al., 2016). One week later, these rats underwent CL to induce HF. After confirming a large myocardial infarction by echocardiogram within 24 h following the CL surgery, these rats were implanted with an osmotic minipump (Alzet Osmotic Pump, Model# 2004, 0.25 μl/h) for a 4-week intracerebroventricular (ICV) infusion of the TACE inhibitor TAPI-0 (10 µg/day, n = 17) or vehicle (VEH, 20% DMSO, n = 18). One rat with a small myocardial infarction was excluded from further study. Seven HF rats died before the end of the protocol. The surviving HF animals were assigned to two experimental groups: HF+TAPI-0 (*n* = 14) and HF+VEH (*n* = 13).

After 4-week treatments with TAPI-0 and VEH, the same procedures for assessment of left ventricular function, hemodynamic measurements and sample collections described in Protocol I were performed. The experimental procedures in this protocol are summarized in Figure 1B.

## HF induction and echocardiography

Rats were anesthetized (ketamine 100 mg/kg and xylazine 10 mg/kg ip) and underwent surgery under aseptic conditions to ligate the left coronary artery to induce HF, or an identical surgical procedure without ligating the left coronary artery to produce SHAM rats, as previously described (Yu et al., 2017; 2019b). Within 24 h and 4 weeks after ligation of the coronary artery, under ketamine sedation (60 mg/kg ip), the left ventricular ejection fraction (LVEF), ischemic zone as a percent of LV circumference (% IZ), LV end-systolic volume (LVESV) and LV end-diastolic volume (LVEDV) were measured by echocardiography as previously described (Yu et al., 2017; 2019b).

## Cardiac hemodynamic and anatomical assessments

At the termination of the study protocols, rats were anesthetized with urethane (1.5 g/kg ip) and a Millar catheter was inserted into the LV *via* the right carotid artery. Hemodynamic parameters including blood pressure, heart rate (HR), LV peak systolic pressure, LV end-diastolic pressure (LVEDP), and maximum rate of rise of LV pressure (LV dP/dt _max_) were measured as previously described (Yu et al., 2017; 2019b). At the end of the experiments, heart and lung were collected and weighed. The heart weight-to-body weight (BW) and wet lung weight-to-BW ratios were determined as indicators of cardiac remodeling and pulmonary congestion.

### Surgical preparation for ICV cannulation and bilateral PVN microinjection

*Implantation of cerebroventricular cannulas:* Intracerebroventicular injection was performed as previously described (Yu et al., 2019a; Yu et al., 2022a). Briefly, under anesthesia with ketamine plus xylazine (100 mg/kg ± 10 mg/kg ip), the animal was fixed in a stereotaxic apparatus (Kopf Instruments, Tujunga, CA). Under aseptic conditions, the skull was exposed and a small hole was drilled to facilitate placement of a 25-gauge stainless steel guide cannula just above the left lateral cerebral ventricle (stereotaxic coordinates AP, -1.0 mm; DV, -4.0 mm; and ML, -1.5 mm, with bregma as a reference). The cannula was secured in place with three protective screws and dental orthodontic resin was applied to the surface of the skull. A 31-gauge stainless steel cannula inserted into the guide cannula and advanced 0.5 mm beyond its tip was used for infusion of drug or VEH into the left lateral cerebral ventricle.

*Implantation of osmotic minipumps:* Rats were anesthetized with ketamine plus xylazine (100 mg/kg ± 10 mg/kg ip). Under aseptic conditions, an osmotic mini-pump (model 2004, Alzet, CA) containing drug or VEH was implanted subcutaneously at the back of the neck and connected to the cannula implanted in the lateral cerebral ventricle. At the end of the study, the osmotic mini-pump was removed to check residual volume of the drug to ensure that all the drug was infused into the animals.

*Preparation for bilateral microinjection into PVN:* Bilateral PVN microinjections of a TACE siRNA (0.15 µl of 10^13^ GC/ml per side) or a scrambled siRNA adeno-associated virus 9 vector encoding green fluorescent protein was performed as previously described (Yu et al., 2022a). Briefly, the skull was exposed under a longitudinal skin incision, and two small holes were drilled at 1.8 mm posterior to bregma and 0.4 mm from midline. A 29-gauge guide cannula was inserted to a position 5.8 mm ventral to dura and a 35-gauge (128 μm outer diameter, and 51.2 μm inner diameter) injection cannula connected to a 1.0 µl micro-syringe was inserted into the guide cannula. The tip of the injection cannula was adjusted to a length extending 2 mm beyond the tip of the guide cannula. Microinjections of TACE siRNA and Scr siRNA AAV vectors in bilateral PVN were made over 30 s simultaneously using 1 μl Hamilton microsyringes. The microinjection sites in the PVN were verified by GFP expression. According to previous report and our prior studies (Yang et al., 2012; Yu et al., 2022a), AAV9 siRNA-mediated gene silencing in peripheral tissue and the brain can last at least 4 weeks after delivery.

### Molecular studies

*Tissue preparations:* Brains and hearts were quickly removed. The PVN regions, including small amounts of surrounding tissues, were punched from brain with a 15-gauge needle stub (inner diameter: 1.5 mm). The peri-infarct area of LV was excised from the heart and frozen in liquid nitrogen for RNA and protein preparation. Total RNA was extracted from PVN punches and peri-infarct area of LV with the RNeasy^®^ Plus Mini Kit (QIAGEN, Germantown, MD). Total protein was extracted from PVN punches using N-PER Neuronal Protein Extraction Reagent (Thermo Fisher Scientific, Rockford, IL).

*Real-Time PCR:* mRNA expression for inflammatory markers TACE, TNF-α, TNFR1, TNF-α receptor 2 (TNFR2), cyclooxygenase (COX)-2 and COX-1, and neuronal activity marker c-Fos in the PVN, and mRNA expression for pro-fibrotic markers α-smooth muscle actin (α-SMA), transforming growth factor- β1 (TGF-β1), fibronectin, collagen-I and collagen-III, and pro-hypertrophic markers atrial natriuretic peptide (ANP), brain natriuretic peptide (BNP), and β-myosin heavy chain (β-MHC) in the peri-infarct area of LV, were analyzed with SYBR Green real-time PCR after reverse transcription of total RNA as previously described. Sequences for each primer pair are shown in Table 1. The QuantStudio™ 3 Real-Time PCR System (Applied Biosystems, Carlsbad, CA) was used to perform real-time PCR. mRNA data were corrected by β-actin and expressed as fold changes relative to the control group.

**TABLE 1:** Sequences for primers.

*Gene*	*Primers*	*Sequences*
TACE	Forward primer: Reverse primer:	5′-GCA​CAG​GGA​ATA​GCA​GTG​AG-3′ 5′-CAC​GAG​TTG​TCG​GTG​TCA​G-3′
TNF-α	Forward primer: Reverse primer:	5′-CCT​TAT​CTA​CTC​CCA​GGT​TCT​C-3′ 5′-TTT​CTC​CTG​GTA​TGA​ATG​GC-3′
TNFR1	Forward primer: Reverse primer:	5′-GGT​TCC​TTT​GTG​GCA​CTT​GGT-3′ 5′-CTC​TTG​GTG​ACC​GGG​AGA​AG-3′
TNFR2	Forward primer: Reverse primer:	5′-TAG​GAC​TGG​CGA​ACT​GCT​T-3′ 5′-AAC​TGG​GTG​CTG​TGG​TCA​AT-3′
COX-2	Forward primer: Reverse primer:	5′-AAG​GGA​GTC​TGG​AAC​ATT​GTG​AAC-3′ 5′-CAA​ATG​TGA​TCT​GGA​CGT​CAA​CA-3′
COX-1	Forward primer: Reverse primer:	5′-AGA​GAT​CAC​CAA​TGC​CAG​CT-3′ 5′-ACT​GGA​TGG​TAC​GCT​TGG​TC-3′
c-Fos	Forward primer: Reverse primer:	5′-GTC​AAC​ACA​CAG​GAC​TTT​TGC​G-3′ 5′-CGT​GGG​GAT​AAA​GTT​GGC​ACT-3′
ANP	Forward primer: Reverse primer:	5′-GCC​GGT​AGA​AGA​TGA​GGT​CA-3′ 5′-GGG​CTC​CAA​TCC​TGT​CAA​TC-3′
BNP	Forward primer: Reverse primer:	5′-TCT​GCT​CCT​GCT​TTC​CTT​A-3′ 5′-GGA​CTA​TGT​GCC​ATC​TTG​GA-3′
β-MHC	Forward primer: Reverse primer:	5′-GCC​AAC​ACC​AAC​CTG​TCC​AAG​TTA-3′ 5′-TTC​AAA​GGC​TCT​CCA​GGT​CTC​AGG​GC-3′
TGF-β1	Forward primer: Reverse primer:	5′-TAT​AGC​AAC​AAT​TCC​TGG​CG-3′ 5′-TGC​TGT​CAC​AGG​AGC​AGT​G-3′
collagen I	Forward primer: Reverse primer:	5′-GAG​GGC​GAG​TGC​TGT​CCT​T-3′ 5′-GGT​CCC​TCG​ACT​CCT​ATG​ACT​TC-3′
collagen III	Forward primer: Reverse primer:	5′-TGA​AGG​AAA​TAG​GAA​ATT​CAC​TTA​CAC-3′ 5′-TCA​AAG​ACT​GTC​TTG​CTC​CAT​TC-3′
α-SMA	Forward primer: Reverse primer:	5′-TTC​GTT​ACT​ACT​GCT​GAG​CGT​GAG A-3′ 5′-AAA​GAT​GGC​TGG​AAG​AGG​GTC-3′
Fibronectin	Forward primer: Reverse primer:	5′-GCT​GCT​GGG​ACT​TCC​ACG​T-3′ 5′-TCT​GTT​CCG​GGA​GGT​GCA-3′
β-actin	Forward primer: Reverse primer:	5′-CCG​CGA​GTA​CAA​CCT​TCT-3′ 5′-CGT​CAT​CCA​TGG​CGA​ACT-3′

*Western blot:* Protein levels for TACE, phosphorylated (p-) and total extracellular signal-regulated protein kinases 1 and 2 (ERK1/2), phosphorylated (p-) and total nuclear factor kappa B p65 (NF-κB p65), NF-κB inhibitor-α (IκB-α), and β-actin in the PVN were determined by Western blot analysis. Briefly, protein samples were separated by 10% SDS-PAGE and then transferred to polyvinylidene difluoride membranes. The membrane was incubated with primary antibodies to TACE (LSBio, Seattle WA), p-ERK1/2 and total ERK1/2, p-NF-κB p65 and total NF-κB p65, IκB-α, and β-actin (Cell Signaling Technology, Danvers, MA) followed by horseradish peroxidase secondary antibodies (Santa Cruz Biotechnology, Santa Cruz, CA). The density of the Western bands was detected and quantified with Image Lab analysis software (Bio-Rad, Hercules, CA).

### Fluorescent studies

To verify the transfection potential of the siRNA virus in the PVN, animals treated with bilateral PVN microinjection of a Scr or TACE siRNA were euthanized by decapitation. The brains were removed rapidly and frozen immediately in liquid nitrogen. The brain regions including PVN were cut into 16 μm coronal sections with a cryostat. GFP tagged in the AAV9 was visualized by Fluorescence Microscopy.

### Histological studies

To examine the LV remodeling, heart was embedded in OCT and snap-frozen on mixture of acetone and dry ice. The heart was horizontally cut into 7 μm sections at the level of the papillary muscle with a cryostat. The heart sections were fixed in 10% buffered formalin and then stained with Masson’s Trichrome for fibrosis or wheat germ agglutinin for cardiomyocyte hypertrophy. The percent fibrosis and cross-sectional area of cardiomyocytes in the peri-infarct area of LV were measured and quantified using ImageJ software as previously described (Dick et al., 2019).

### Biochemical assays

*CSF collection:* The CSF was collected from the cisterna magna. Under anesthesia with urethane (1.5 g/kg ip), rats were placed in a stereotaxic frame and secured with ear bar. The rat skull was positioned downward with a 45° degree. One midline incision was made to expose the atlanto-occipital membrane. The CSF was withdrawn from the cisterna magna using a 26 G needle connected with 1-ml syringe. The color of the CSF samples was closely observed to avoid any possible blood contamination.

CSF and trunk blood were collected at the time of euthanasia for biochemical assays. CSF levels of TNF-α and plasma levels of norepinephrine (NE, an indicator of global sympathetic activity) were measured with commercial ELISA kits (R&D Systems, Minneapolis, MN and LSBio, Seattle, WA, respectively) according to the manufacturers’ instructions.

### Statistical analysis

All data are shown as mean ± SEM. All molecular, biochemical and histologic measurements were performed blindly. Normal distributions and equal variances were checked using Kolmogorov-Smirnov test and Levene’s test, respectively. Statistical analyses were performed using Student’s t-test, one-way or two-way ANOVA followed by Tukey’s multiple comparison tests. *p* < 0.05 was considered statistically significant.

## Results

### Validation of tumor necrosis factor-α converting enzyme knockdown by its siRNA in the paraventricular nucleus

**FIGURE 2:**
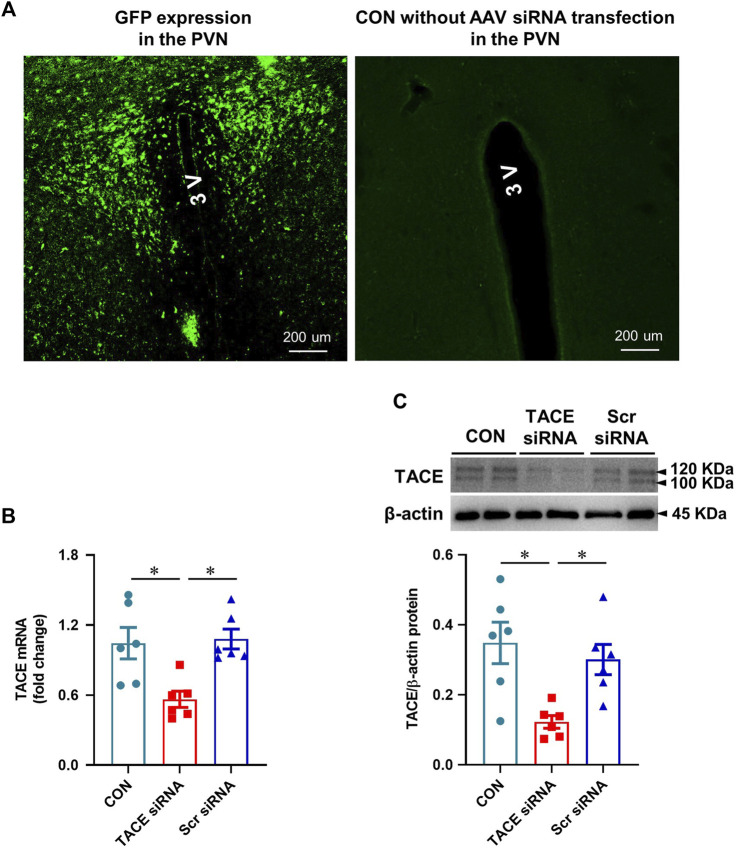
Validation of PVN transfection and TACE knockdown. **(A)**: Expression of green fluorescent protein (GFP) in the PVN in a normal rat 1 week after bilateral PVN microinjections of an adeno-associated virus (AAV) vector encoding TACE siRNA and GFP and a control rat without treatment with AAV vector. GFP was distributed diffusely throughout the PVN. **(B and C)**: mRNA and protein expression of TACE in the PVN of rats 1 week after PVN microinjections of TACE siRNA or scrambled (Scr) siRNA. Non-treated normal rats served as control (CON). Values are mean ± SEM (n = 6 for each group). One-way ANOVA followed by Tukey’s post hoc tests was used for data analysis. mRNA data are expressed as a fold change compared to CON. *p < 0.05.

One week after bilateral PVN microinjections of the AAV vector carrying TACE siRNA, GFP fluorescence was present throughout the PVN in normal animals, demonstrating effective viral transfection of the PVN (Figure 2A). Real-time PCR and Western blot analysis revealed significantly lower TACE mRNA expression (Figure 2B) and protein levels (Figure 2C) in the PVN of animals treated with TACE siRNA, but not the animals treated with Scr siRNA when compared with the measurements in untreated control rats. These observations confirmed the accuracy of the microinjection sites and the efficacy of genetic knockdown of TACE in the PVN with a siRNA AAV specifically targeting TACE.

### Effect of paraventricular nucleus tumor necrosis factor-α converting enzyme knockdown on the local expression of inflammatory mediators in HF

**FIGURE 3:**
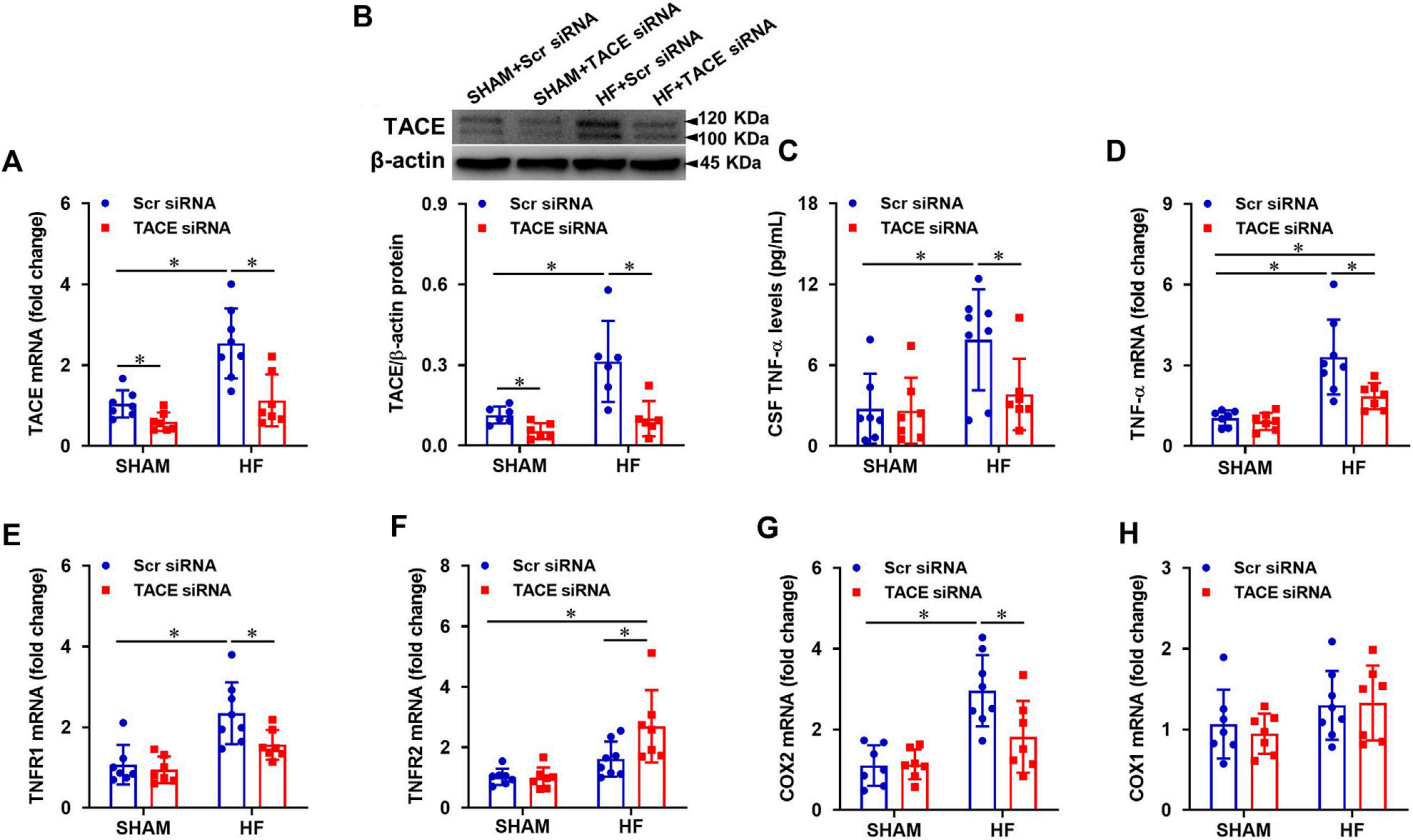
mRNA and protein levels of TACE in the PVN **(A and B)**, soluble tumor necrosis factor (sTNF)-α levels in the cerebrospinal fluid (CSF, **(C)**, and mRNA expression of inflammatory mediators TNF-α **(D)**, TNF-α receptor 1 (TNFR1, **(E)**, TNFR2 **(F)**, cyclooxygenase (COX)-2 **(G)** and COX-1 **(H)** in the PVN in HF or SHAM rats pretreated with PVN microinjection of TACE siRNA or Scr siRNA. Values are mean ± SEM (n = 6-8 for each group). Two-way ANOVA followed by Tukey’s post hoc tests was used for data analysis. mRNA data are expressed as a fold change compared to SHAM + Scr siRNA. *p < 0.05.

Four weeks after CL, mRNA expression (Figure 3A) of TACE in the PVN were markedly higher in HF + Scr siRNA than those in SHAM + Scr siRNA rats. Similarly, the protein levels of TACE in both mature (≈100 kDa) and immature (≈120 kDa) forms (Figure 3B) were also elevated in HF + Scr siRNA vs SHAM + Scr siRNA rats. TACE siRNA treatment significantly reduced mRNA expression and protein levels of TACE in the PVN in both SHAM and HF animals. The protein levels of TNF-α in the CSF (Figure 3C) were also significantly elevated in HF + Scr siRNA rats, along with increased mRNA expression of TNF-α (Figure 3D), TNFR1 (Figure 3E), and COX-2 (Figure 3G) in the PVN compared with SHAM + Scr siRNA rats. However, the mRNA expression of TNFR2 in the PVN in HF + Scr siRNA rats did not differ significantly from that in SHAM + Scr siRNA rats (Figure 3F). Bilateral PVN microinjection of TACE siRNA significantly reduced the protein levels of TNF-α in the CSF, the mRNA expression of TNF-α, TNFR1, and COX-2, and augmented mRNA level of TNFR2 in the PVN in HF rats. Treatment with TACE siRNA did not alter the expression of these inflammatory mediators in SHAM rats. The COX-1 mRNA expression (Figure 2H) in the PVN had no change across the four experimental groups.

### Effect of genetic knockdown of tumor necrosis factor-α converting enzyme in the paraventricular nucleus on the local sympatho-excitatory mediators

**FIGURE 4:**
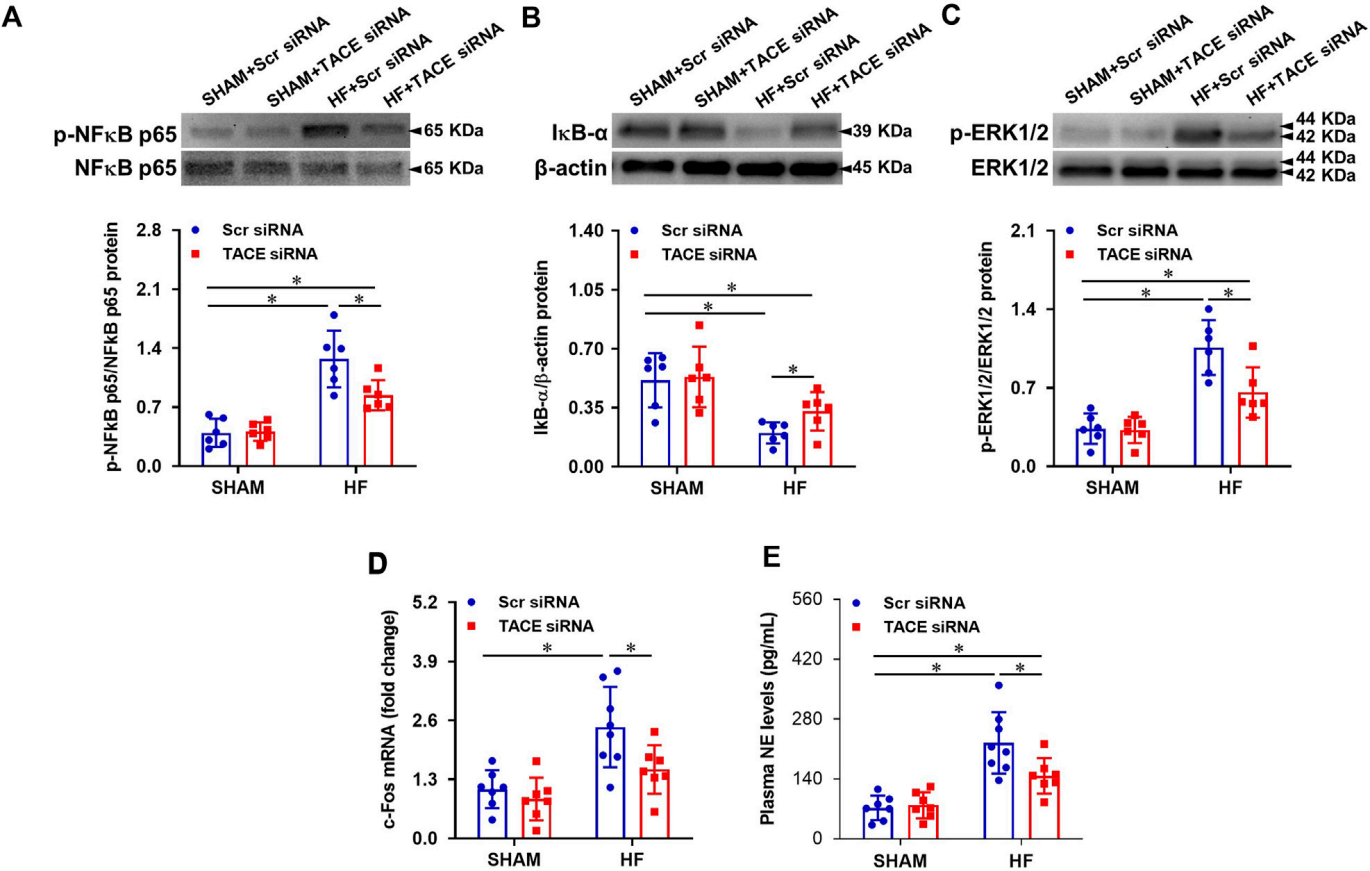
Protein levels of phosphorylated (p-) NF-κB p65 **(A)** and its inhibitor IκB-α **(B)**, p-ERK1/2 **(C)**, and mRNA expression of c-Fos (a marker of neuronal activity, **(D)** in the PVN, and levels of plasma norepinephrine (NE, a marker of sympathetic nerve activity, **(E)**, in HF or SHAM rats pretreated with PVN microinjection of TACE siRNA or Scr siRNA. Values are mean ± SEM (n = 6-8 for each group). Two-way ANOVA followed by Tukey’s post hoc tests was used for data analysis. mRNA data are expressed as a fold change compared to SHAM + Scr siRNA. *p < 0.05.

Compared with SHAM + Scr siRNA rats, HF + Scr siRNA rats had higher protein levels of p-NF-κB p65 (Figure 4A), lower protein levels of IκB-α (Figure 4B), and higher protein levels of p-ERK1/2 (Figure 4C) in PVN. TACE siRNA treatment significantly reversed these changes in the PVN of HF rats but had no effects on these variables in SHAM rats.

mRNA expression of c-Fos (Figure 4D), a recognized marker of neuronal excitation, in the PVN and plasma levels of NE (Figure 4E), an important indicator of sympathetic activity, were increased in the HF + Scr siRNA rats when compared with SHAM + Scr siRNA rats. TACE siRNA treatment reduced HF-induced increases in mRNA expression of c-Fos and plasma NE levels. There were no differences in mRNA expression of c-Fos and plasma NE levels between the two SHAM groups.

### Effect of genetic knockdown of tumor necrosis factor-α converting enzyme in the paraventricular nucleus on cardiac function and hemodynamics in HF

**FIGURE 5:**
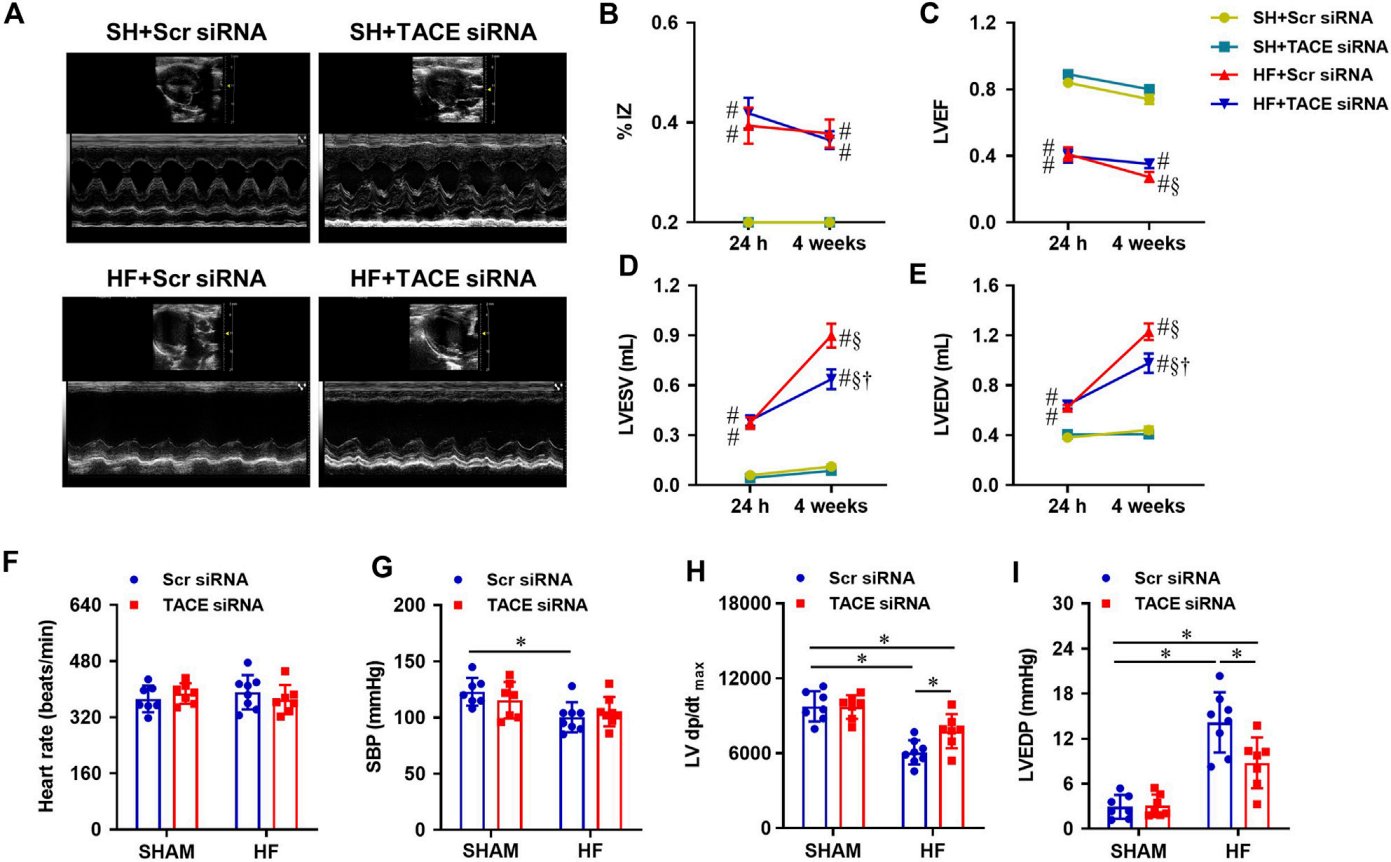
**(A)** Representative M-mode echocardiographic images from HF or SHAM rats pretreated with PVN microinjection of TACE siRNA or Scr siRNA (4 weeks after coronary artery ligation). **(B–E)**: Quantitative comparison of echocardiographic parameters including ischemic zone as a percent of left ventricular (LV) circumference (% IZ), LV ejection fraction (LVEF), LV end-systolic volume (LVESV) and LV end-diastolic volume (LVEDV) 24 h and 4 weeks after coronary artery ligation. **(F–I)**: Quantitative comparison of LV hemodynamic parameters including heart rate, systolic blood pressure (SBP), maximum rate of rise of LV pressure (LV dP/dt_max_) and LV end-diastolic pressure (LVEDP) from four treatment groups 4 weeks after coronary artery ligation. Values are mean ± SEM (n = 7-8 for each group). Two-way ANOVA followed by Tukey’s post hoc tests was used for data analysis. #*p* < 0.05 vs SHAM + Scr siRNA; §*p* < 0.05 vs same group at 24 h; †*p* < 0.05, HF+TACE siRNA vs HF + Scr siRNA; *p < 0.05.

Prior to the treatments, echocardiography performed within 24 h of CL revealed that the infarct size (% IZ, Figure 5B) and the degree of cardiac dysfunction as indicated by LVEF (Figure 5C), LVESV (Figure 5D) and LVEDV (Figure 5E), were similar in both HF + Scr siRNA and HF + TACE siRNA groups. After the 4-week treatment protocol, the infarct size (%IZ) was unchanged in HF + Scr siRNA rats and HF + TACE siRNA rats. However, cardiac function in the HF + Scr siRNA rats was further compromised with decreased LVEF and increased LVESV and LVEDV. The 4-week treatment with TACE siRNA prevented the reduction of LVEF in the HF rats and lessened the further increment of LVESV and LVEDV. Echocardiographic indexes of cardiac function were similar between the two SHAM groups.

Hemodynamic measurements showed that HR (Figure 5F) was comparable across the four experimental groups. Systolic blood pressure (SBP, Figure 5G) and LV dP/dt_max_ (Figure 5H) were lower and LVEDP (Figure 5I) was higher in HF + Scr siRNA rats than SHAM + Scr siRNA rats. HF + TACE siRNA rats exhibited similar SBP but had higher LV dP/dt_max_ and lower LVEDP compared with HF + Scr siRNA rats. Of note, the values for LV dP/dt_max_ and LVEDP were still significantly different from those in SHAM + Scr siRNA rats. TACE siRNA treatment did not affect the hemodynamic parameters in SHAM rats.

### Effect of genetic knockdown of tumor necrosis factor-α converting enzyme in the paraventricular nucleus on anatomic indicators of HF

**FIGURE 6:**
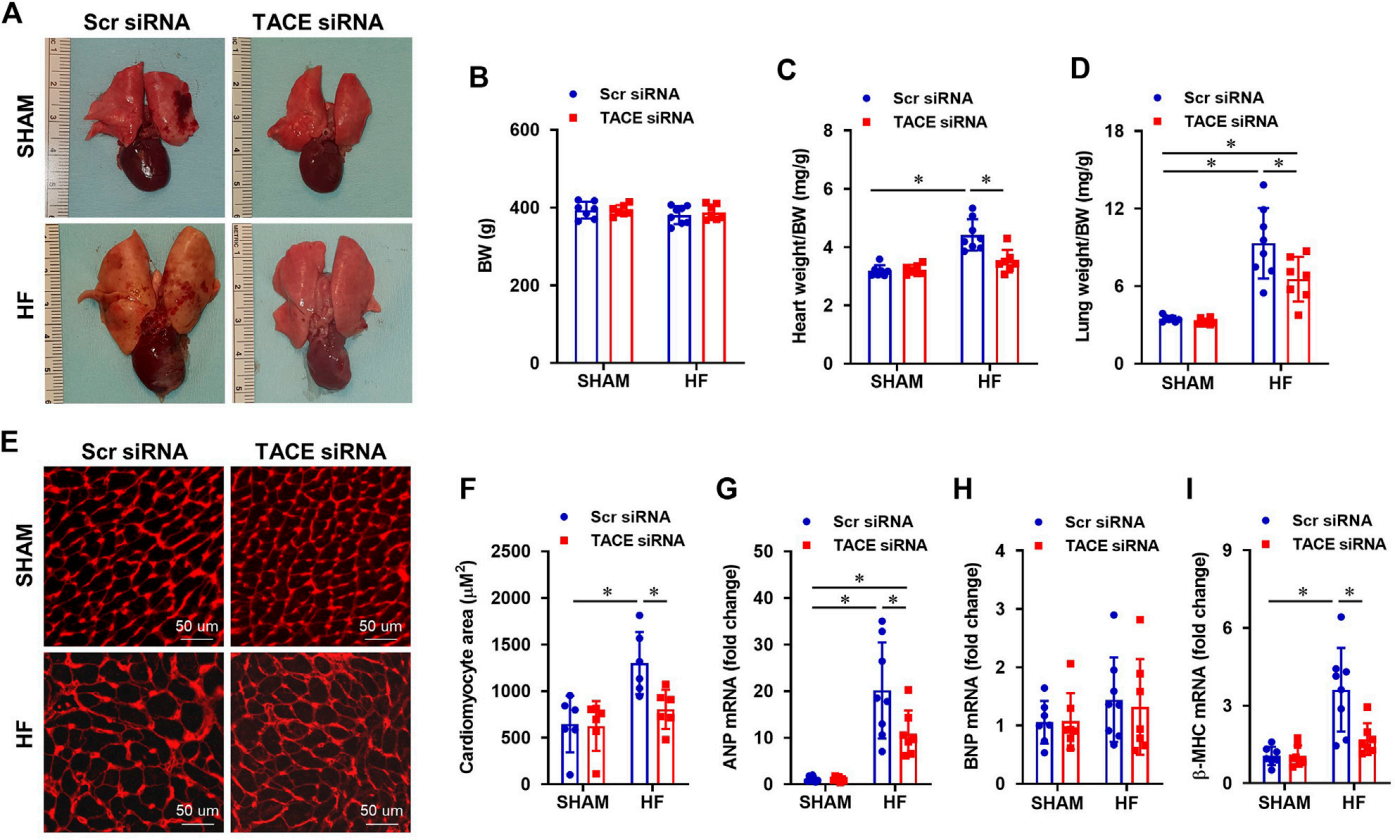
**(A)** Representative heart and lung images from HF or SHAM rats pretreated with PVN microinjection of TACE siRNA or Scr siRNA (4 weeks after coronary artery ligation). **(B–D)**: Anatomic measurements including body weight (BW), ratios of heart weight or lung weight to BW. **(E)** Representative wheat-germ-agglutinin staining (WGA) showing cardiomyocyte area from four treatment groups. **(F–I)**: Quantitative comparison of the cross-sectional area of myocytes and mRNA expression of pro-hypertrophic markers atrial natriuretic peptide (ANP), brain natriuretic peptide (BNP) and β-myosin heavy chain (β-MHC), in the peri-infarct areas of the left ventricular in treatment groups. Two-way ANOVA followed by Tukey’s post hoc tests was used for data analysis. Values are mean ± SEM (n = 6-8 for each group). *p < 0.05.

The ratios of heart-to-BW and lung-to-BW were used to evaluate cardiac remodeling and pulmonary congestion, respectively. Figure 6A shows typical images of the lung and heart taken from each treatment group at the end of experiments. Although BW was similar between groups (Figure 6B), the ratios of heart-to-BW (Figure 6C) and wet lung-to-BW (Figure 6D) were substantially higher in HF + Scr siRNA rats compared with SHAM + Scr siRNA rats. Treatment with TACE siRNA markedly improved the heart-to-BW and wet lung-to-BW ratios in HF, but not the SHAM rats.

**FIGURE 7:**
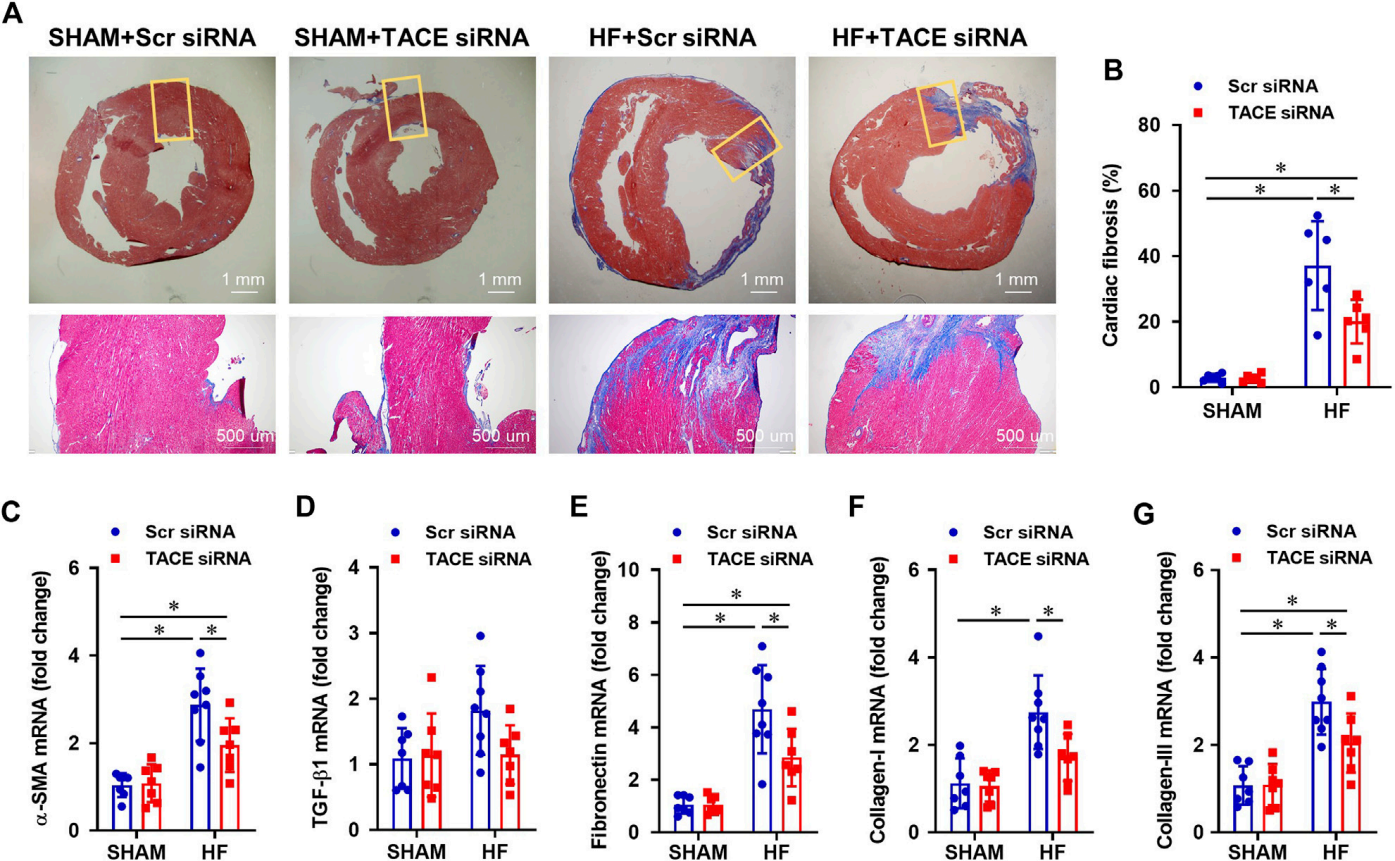
**(A)** Representative Masson’s Trichrome staining showing cardiac fibrosis from HF or SHAM rats pretreated with PVN microinjection of TACE siRNA or Scr siRNA (4 weeks after coronary artery ligation). **(B–G)**: Quantitative comparison of the collagen deposition and mRNA expression of pro-fibrotic markers α-smooth muscle actin (α-SMA), transforming growth factor (TGF)-β1, fibronectin, collagen-I and collagen-III, in the peri-infarct areas of the left ventricular in four treatment groups. Values are mean ± SEM (n = 6-8 for each group). Two-way ANOVA followed by Tukey’s post hoc tests was used for data analysis. *p < 0.05.

Histological analysis showed that the cross-sectional area of myocytes (Figures 6E,F) and collagen deposition (Figures 7A,B), indicators of cardiomyocyte hypertrophy and cardiac fibrosis, respectively, were robustly increased in the peri-infarct area of LV in HF + Scr siRNA rats compared with SHAM + Scr siRNA rats. These observations were accompanied with upregulated mRNA expression of pro-hypertrophic markers ANP (Figure 6G) and β-MHC (Figure 6I), and pro-fibrotic markers α-SMA (Figure 7C), fibronectin (Figure 7E), collagen-I (Figure 7F) and collagen-III (Figure 7G). TACE siRNA treatments significantly attenuated the cross-sectional area of myocytes and the collagen deposition as well as the mRNA expression of ANP, β-MHC, α-SMA, fibronectin, collagen-I and collagen-III in HF rats but not in SHAM rats. There was no significant difference in mRNA expression of pro-hypertrophic marker BNP (Figure 6H) and pro-fibrotic marker TGF-β1 (Figure 7D) across the four experimental groups in either HF or SHAM rats.

### Effect of central inhibition of tumor necrosis factor-α converting enzyme activity on inflammatory and sympatho-excitatory mediators in HF

**FIGURE 8:**
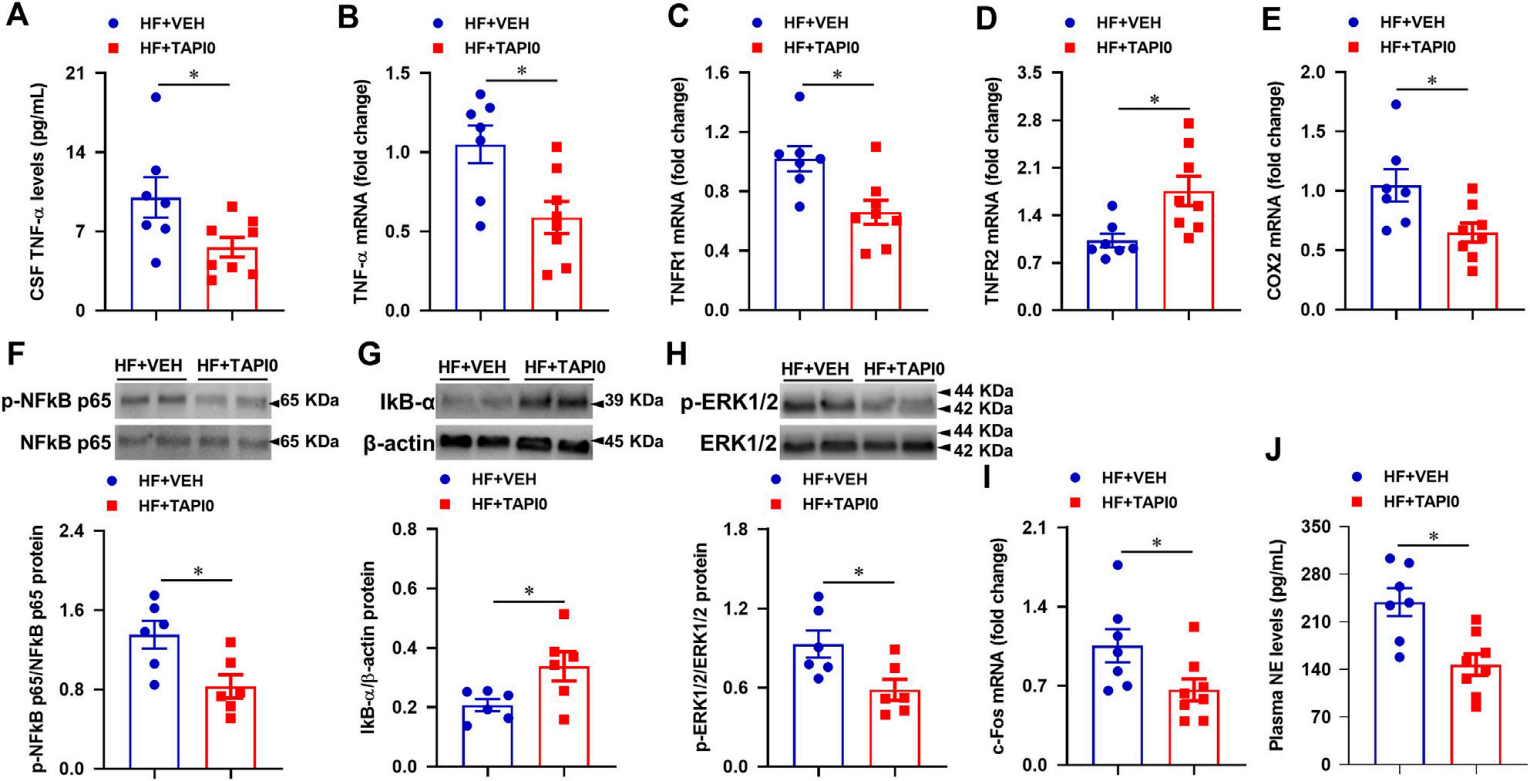
Quantitative comparison of the levels of inflammatory mediators including TNF-α in CSF **(A)**, mRNA of TNF-α **(B)**, TNFR1 **(C)**, TNFR2 **(D)** and COX-2 **(E)**, protein of p-NF-κB p65 **(F)** and its inhibitor IκB-α **(G)**, and p-ERK1/2 **(H)**, and mRNA of neuronal excitatory marker c-Fos **(I)** in the PVN, and levels of sympathetic excitatory marker norepinephrine (NE, **(J)** in plasma, in HF rats treated with a 4-week ICV TACE inhibitor TAPI-0 or vehicle (VEH). Values are mean ± SEM (*n* = 6-8 for each group). Student’s t-test was used for data analysis. mRNA data are expressed as a fold change compared to HF+VEH. *p < 0.05.

To verify the effects of genetic knockdown of TACE in the PVN in HF, we further performed a 4-week ICV intervention to inhibit central TACE activity with its inhibitor TAPI-0. TAPI-0 acts as a pseudosubstrate binding with catalytic zinc element at the active center of TACE to inhibit its catalytic activity (Fan et al., 2003). Compared with HF + VEH rats, HF + TAPI-0 rats had significantly lower levels of TNF-α in CSF (Figure 8A), mRNA expression of TNF-α (Figure 8B), TNFR1 (Figure 8C), COX2 (Figure 8E), protein levels of p-NF-κB p65 (Figure 8F) and p-ERK1/2 (Figure 8H), and higher mRNA expression of TNFR2 (Figure 8D) and protein levels of IκB-α (Figure 8G) in the PVN. Similar to the genetic knockdown of TACE in the PVN, mRNA expression of c-Fos (Figure 8I) and plasma levels of NE (Figure 8J) were also reduced in HF rats treated with TAPI-0 compared with VEH treated HF rats.

Since genetic knockdown of TACE in the PVN by TACE siRNA had no effects on inflammatory mediators, cardiac hemodynamic parameters and anatomic indicators in SHAM rats, to reduce animal usage inhibition of central TACE activity with its inhibitor TAPI-0 was performed only in HF animals in this experimental protocol.

### Effect of central inhibition of tumor necrosis factor-α converting enzyme activity on left ventricular dysfunction in HF

**FIGURE 9:**
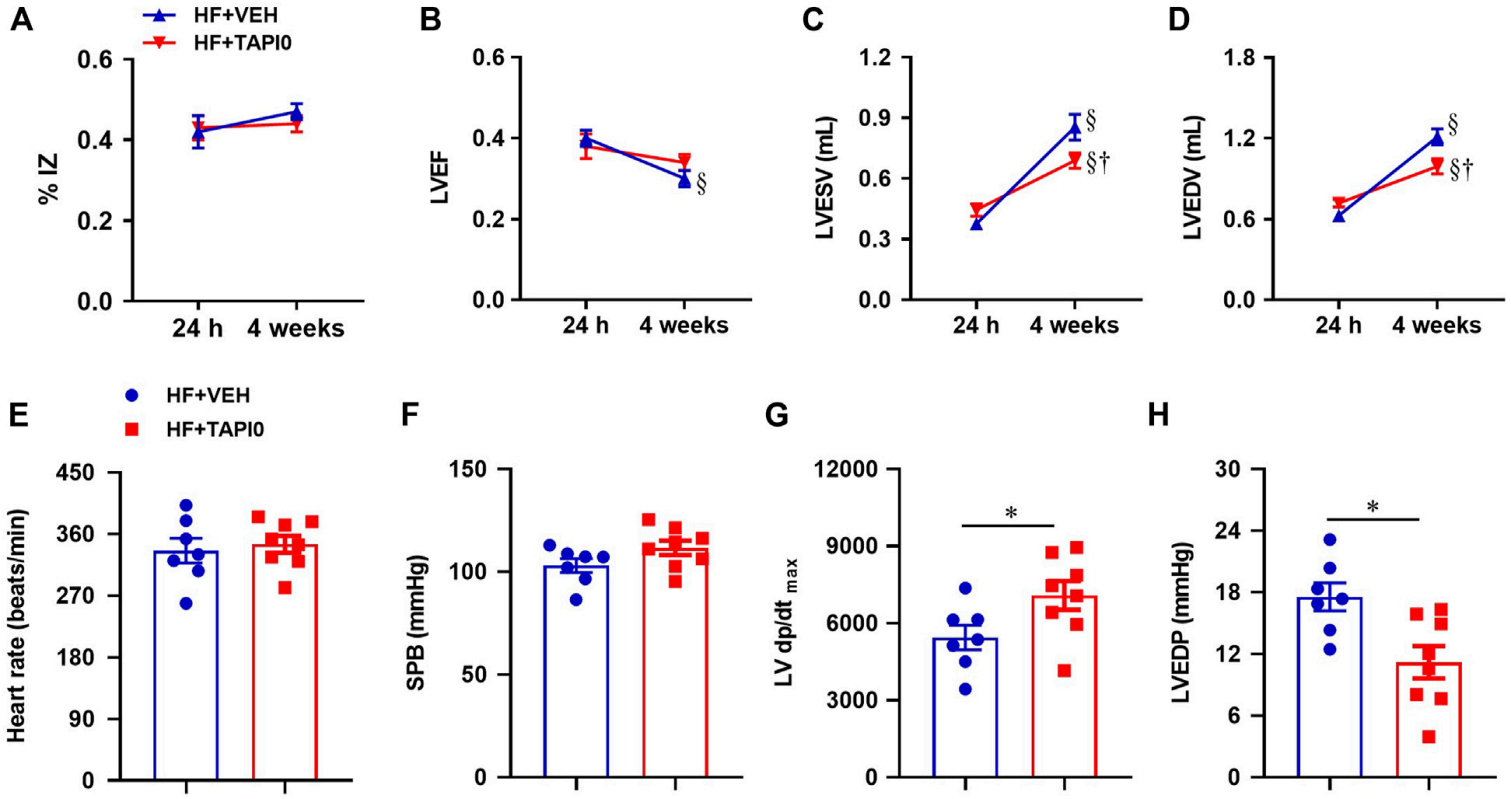
Quantitative comparison of echocardiographic parameters including ischemic zone as a percent of left ventricular (LV) circumference (% IZ, **(A)**), LV ejection fraction (LVEF, **(B)**), LV end-systolic volume (LVESV, **(C)**) and LV end-diastolic volume (LVEDV, **(D)**), and LV hemodynamic parameters including heart rate **(E)**, systolic blood pressure (SBP, **(F)**), maximum rate of rise of LV pressure (LV dP/dt_max_, **(G)**) and LV end-diastolic pressure (LVEDP, **(H)**), from HF rats treated with TACE inhibitor TAPI-0 or vehicle (VEH). Values are mean ± SEM (n = 7-8 for each group). Two-way ANOVA followed by Tukey’s post hoc tests was used for echocardiography data analysis. Student’s t-test was used for hemodynamic data analysis. §*p* < 0.05 vs same group at 24 h; †*p* < 0.05, HF+TAPI0 vs HF+VEH; *p < 0.05.

Echocardiography within 24 h after CL, revealed a large myocardial infarction (ischemic zone as a percent of LV circumference, %IZ, Figure 9A) and cardiac dysfunction as measured by LV ejection fraction (LVEF, Figure 9B), LV end systolic volume (LVESV, Figure 9C) and LV end diastolic volume (LVEDV, Figure 9D) in HF rats assigned to treatment with TAPI-0 or VEH. After a four-week ICV treatment, %IZ remained unchanged in both HF groups. While the low LVEF of the HF rats measured within 24 h decreased further after a 4-week treatment with ICV VEH, LVEF did not decrease further in the TAPI-0-treated HF rats at 4 weeks. Compared with the echocardiographic measurements within 24 h, LVESV and LVEDV had increased further at 4 weeks in both HF groups, but with a milder increment in HF + TAPI-0 rats.

Heart rate (Figure 9E) and systolic blood pressure (Figure 9F) were comparable in HF rats treated with TAPI-0 or vehicle (VEH), but the maximum rate of rise of LV pressure LV dP/dt max (Figure 9G) was higher and LV end-diastolic pressure (Figure 9H) was lower in HF + TAPI-0 rats than HF + VEH rats.

### Effect of central inhibition of tumor necrosis factor-α converting enzyme activity on cardiac remodeling in HF

**FIGURE 10:**
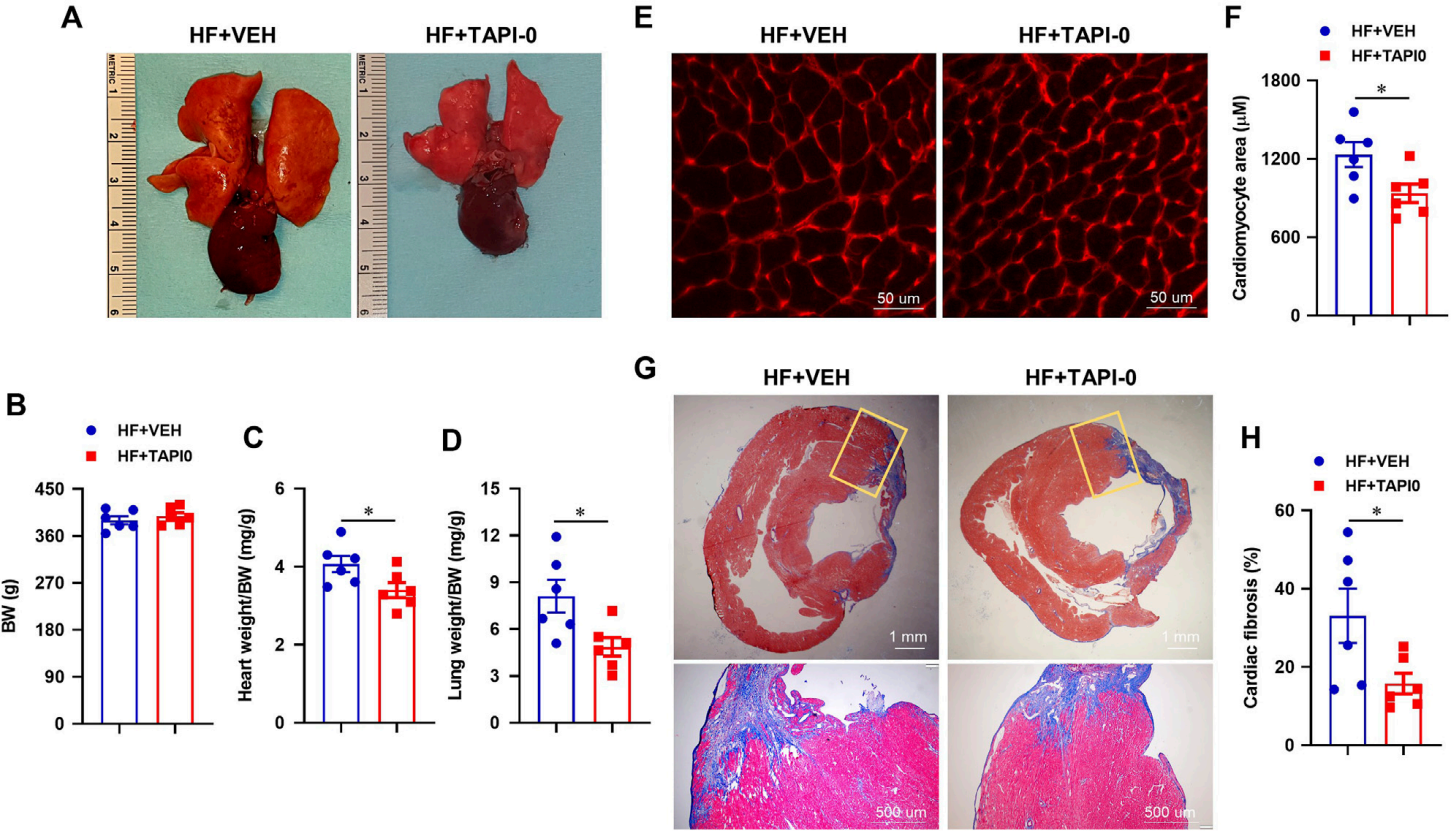
Representative heart and lung images **(A)** and anatomic measurements **(B,C and D)** including body weight (BW), ratios of heart weight or lung weight to BW from HF rats treated with TACE inhibitor TAPI-0 or vehicle (VEH). **(E and F)** Representative wheat-germ-agglutinin staining (WGA) showing cardiomyocyte area and quantitative comparison of the cross-sectional area of myocytes in the peri-infarct areas of the left ventricular in the two treatment groups. **(G and H)** Representative Masson’s Trichrome staining showing cardiac fibrosis and quantitative comparison of the collagen deposition in the peri-infarct areas of the left ventricular in the two treatment groups. Student’s t-test was used for data analysis. Values are mean ± SEM (n = 6 for each group). *p < 0.05.

Compared with HF rats treated with VEH, the morphological images (Figure 10A) showed a reduced size of lung and the heart in HF rats treated with a 4-week ICV TAPI-0. There was no significant difference in body weight between the two HF treatment groups (Figure 10B). Like rats treated with genetic knockdown of TACE in the PVN, HF + TAPI-0 rats had lower ratios of heart-to-body weight (Figure 10C) and wet lung-to-body weight (Figure 10D), compared with HF + VEH rats.

Additionally, the cross-sectional area of myocytes (Figures 10E,F) and collagen deposition (Figures 10G,H) in the peri-infarct area of the failing heart were significantly decreased in HF + TAPI-0 rats compared with HF + VEH rats.

## Discussion

As an essential modulator in TNF-α-induced inflammatory responses, TACE is emerging as a potential molecular target in the treatment of cardiovascular diseases (Takayanagi et al., 2016; Kawai et al., 2021). A number of preclinical and clinical studies in chronic HF disease model suggest that targeting TACE may be an attractive strategy to slow the progression and attenuate the severity of this devastating disease. In this work, we focused on targeting TACE activity in the brain as a potential therapeutic approach to countering neuroinflammation and neurohumoral activation in HF. Using a rat model of HF induced by myocardial infarction, we found that genetic knockdown of TACE expression in the PVN or inhibition of TACE activity in the brain reduces TNF-α levels along with reduced activation of the NF-κB and ERK1/2 signaling, and decreases expression of inflammatory mediators and sympathetic activity, leading to improvements in cardiac remodeling and dysfunction in the development of HF. These findings support the hypothesis that elevated TACE activity in the brain promotes the production of the active soluble form of TNF-α to induce the neuroinflammatory responses that contribute to sympathetic excitation and its adverse influences in HF.

Previous studies from our lab and others have consistently demonstrated that the levels of TNF-α increase in cardiovascular regions of the brain in HF and contribute to augmented sympathetic outflow and its adverse effects (Francis et al., 2004; Kang et al., 2006; Guggilam et al., 2011; Yu et al., 2019b). Experimental interventions that block the central effects of TNF-α consistently improve the peripheral manifestations of HF (Yu et al., 2017). The present study illustrates a potentially important added advantage of targeting TACE to counter the adverse central effects of TNF-α. TNF-α initiates its biological actions by binding to two receptors, TNFR1 and TNFR2, that are expressed in most cell types in peripheral tissues and in the brain. While TNFR1 is preferentially activated by sTNF-α to play a pro-inflammatory and toxic role, the anti-inflammatory and protective effect of tmTNF-α as a ligand that predominantly binds to TNFR2 is widely appreciated (Grell et al., 1995; Grell et al., 1998). Although either form of TNF-α can activate both receptors, the differential binding affinity of sTNF-α and tmTNF-α to these two receptors determines their pathophysiological effects. Blocking TACE activity and the membrane shedding of sTNF-α, and thus reducing the ligand for TNFR1, theoretically permits an increase in the tmTNF-α ligand for TNFR2, shifting the balance towards the beneficial effects of TNF-α. Interestingly, we found that a genetic reduction or a pharmacological inhibition of TACE activity in the PVN of HF rats not only reduced CSF TNF-α, an indicator of sTNF-α, but concomitantly reduced TNFR1 mRNA and increased TNFR2 mRNA expression, favoring the protective TNFR2-mediated effects of TNF-α. These findings provide new evidence that interventions aimed at brain TACE might be a promising strategy for treating cytokine-induced neuroinflammation and sympathetic activation in HF.

The potential beneficial clinical effects of blocking central TACE activity were apparent in this rat model of HF-induced by myocardial infarction. We previously reported that TACE is expressed in both neurons and glial cells in the PVN in SHAM and HF rats (Yu et al., 2019a). The data from HF rats treated with scr-siRNA demonstrate that TACE-mediated TNF-α-induced neuroinflammation plays an essential role in promoting the progression of cardiac remodeling and dysfunction. TACE knockdown in the PVN resulted in an amelioration of this process, evidenced by reductions in LV dilation, heart-to-BW ratio, cross-sectional area of myocytes and mRNA expression of pro-hypertrophic markers, and collagen deposition and mRNA expression of pro-fibrotic markers in the peri-infarct area. LVEF did not increase after a 4-week treatment with TACE siRNA or its inhibitor, as expected in this model of chronic coronary artery occlusion (Yu et al., 2017), but did not decline further as it did in the vehicle-treated HF rats. But despite the persistent reduction in LVEF, HF rats treated with TACE siRNA or its inhibitor had significant improvements in cardiac contractility and function: LV dP/dt_max_ increased and LVEDP, LVEDV and LVESV decreased. The reduced lung-to-BW ratio, indicating an improvement in pulmonary congestion, is consistent with these hemodynamic findings. In a clinical setting, in patients with HF, these cardiovascular effects of reduced central neuroinflammation would like manifest as enhanced functional capacity.

It should be noted that while significant improvement in the cardiac hemodynamic profile was observed, central inhibition of TACE activity did not substantially ameliorate the echocardiographically defined severely reduced LVEF or LV ischemic zone following a large myocardial infarction. The improvement in hemodynamics is most likely a result of chronic reductions in sympathetically-mediated renal sodium and volume retention and vasoconstriction, with accompanying reductions in preload and afterload of the heart.

The mechanisms underlying upregulated TACE in the brain in HF are not yet fully understood. We previously reported that central administration of angiotensin II increases TACE expression in the brain (Yu et al., 2019a). It is well-documented that the renin-angiotensin system (RAS) in the periphery and central nervous system is activated in HF. Angiotensin II–induced cardiac hypertrophy and fibrosis is effectively prevented by knockdown of TACE expression (Wang et al., 2009). Thus, the activated brain RAS might be a potential contributor to upregulated expression of brain TACE in HF. Central administration of the pro-inflammatory cytokine IL-1β also boosted the expression of TACE in the brain, suggesting a feedforward loop by inflammatory cytokines to promote TACE activity.

The inactive rhomboid protein 2 (iRhom2), a proteolytically inactive member of the rhomboid family, may also be involved in the upregulation of TACE by facilitating its trafficking and release in HF. iRhom2 binds immature TACE and promotes its exit from the endoplasmic reticulum to the Golgi apparatus, where immature TACE undergoes furin-mediated maturation and activation (Adrain et al., 2012; Luo and Shu, 2017; Geesala et al., 2019). The failure of TACE to exit the endoplasmic reticulum in the absence of iRhom2 prevents the furin-mediated maturation and trafficking of TACE to the cell surface, the site of TNF-α cleavage (Adrain et al., 2012). Preliminary evidence suggests that brain TACE activity might be regulated by iRhom2 in HF (Yu et al., 2022b).

The increased level of TNF-α in the brain in HF leads to the activation of multiple signaling pathways, particularly to the activation of NF-κB and mitogen-activated protein kinase (MAPK) pathways that are important to TNF-α-induced inflammatory cascades and sympathetic activation (Wei et al., 2008; Diaz et al., 2020; Wei et al., 2021). Increased NF-κB in the PVN plays a major role as a driver of sympathetic excitation in HF (Kang et al., 2011). In the present study, TACE knockdown or central inhibition of TACE activity significantly reduced the increased NF-κB activity in HF rats, as indicated by decreased levels of p-NF-κB p65 and increased levels of IκB-α.

ERK1/2 MAPK activity (p-ERK1/2) was also increased in the HF rats and reduced by the TACE treatments. Brain ERK1/2 signaling has been implicated as a necessary event for the full expression of sympathetic excitation in HF (Wei et al., 2008; Wei et al., 2021). ERK1/2 MAPK signaling is a key downstream mechanism mediating the effects of the epidermal growth factor receptor (EGFR) (Wetzker and Bohmer, 2003). The ability of TNF-α in the brain to promote sympathetic activation and its adverse effects on the progression of HF is largely dependent upon EGFR and ERK1/2 signaling (Wei et al., 2021). Of note, the sheddase TACE/ADAM17 also cleaves transforming growth factor alpha (TGF-α), an endogenous ligand of EGFR, from its membrane-bound precursor (Lee et al., 2003; Sahin et al., 2004). Both TGF-α and EGFR are abundantly expressed and upregulated in the PVN of HF (Yu et al., 2022a). Hence, TGF-α induced EGFR activation of the ERK1/2 signaling pathway may be an additional mechanism mediating TACE-induced sympathetic overactivity and inflammatory responses in the setting of HF. Undoubtedly, TACE in the PVN may promote sympathetic excitation by other mechanisms as well, such as glutamatergic activation (Xu et al., 2019).

Finally, the expression of c-Fos, an indicator of neuronal excitation in the PVN, and plasma levels of NE, a marker of sympathetic nerve activity, were also reduced in HF rats in which TACE activity was reduced. Taken together, these data indicate that key downstream molecular mechanisms mediating central-TNF-α-induced inflammatory and neurohumoral effects in HF rats can be attenuated by suppressing brain TACE expression or activity.

*Limitations of the study:* The present study was conducted in male animals. In our previous study, the sexual dimorphism in the mRNA expression of TNF-α and other inflammatory mediators in the PVN had been found in HF rats (Yu et al., 2019b). Further studies in female animals are warranted to determine whether sex differences exist regarding the expression of TACE in the brain and the effects of central blockade of TACE on TNF-α induced neuroinflammation, sympathetic activation and cardiac dysfunction in HF. It needs to be mentioned, although TACE inhibition in HF was proposed to enhance the level of tmTNF-α, direct evidence for this hypothesis was not provided due to unavailability of the specific antibody targeting this transmembrane form of TNF-α. Likewise, since the antibody we previously used for immunohistochemical localization of c-Fos is no longer commercially available, the present study employed c-Fos mRNA level as an indicator of neuronal activity in the PVN. This approach has been used by other investigators as well (Holzer et al., 2005; Inoue et al., 2021). Finally, in this study elevated levels of inflammatory mediators TNF-α, its receptors and COX-2 in the brain were taken as indicators of degree of neuroinflammation. Since mRNA levels in the PVN have paralleled the protein levels when both were assessed in our previous studies in this chronic HF model (Yu et al., 2012; Yu et al., 2015; Yu et al., 2017, 2019b), only mRNA levels of these inflammatory components were measured in this work. While their levels were not assessed in this study, other inflammatory mediators, including the pro-inflammatory cytokines IL-1β, IL-6, and IL-17A and the chemokines SDF-1, MCP-1 and MIP-1α, are upregulated in the brain and also contribute to neuroinflammation in HF.

## Perspectives

Central treatments targeting TACE in the brain, and more specifically in the PVN, greatly reduced neuroinflammation, relevant transcriptional activity and sympathetic excitation, and thus improved the cardiac dysfunction in HF. PVN is of special interest and would be a key intervention target in the brain to reduce the neurohumoral activation in cardiovascular disorders. It is worth noting that anti-cytokine agents have been the subject of industry-sponsored clinical trials that have not only failed to show benefit but also have been found to induce serious side effects (Anker and Coats, 2002; Chung et al., 2003; Mann et al., 2004). The identification of TACE as a possible novel target in the treatment of HF is particularly significant for its clinical translational potential. Intervention to lessen TACE activity would potentially reduce the production of sTNF-α and therefore decrease the activation of TNFR1 while at the same time raising the level of tmTNF-α and therefore boosting the activation of TNFR2. In terms of our findings, suppressing TACE expression or activity, particularly in the brain might be a promising therapeutic direction in treating HF. Notably, the findings also have important implications for the treatments of other cardiovascular and metabolic disorders like hypertension, diabetes and obesity, in which the central nervous system effects of neuroinflammation have been shown to contribute to the progression of disease.

## Data Availability

The raw data supporting the conclusion of this article will be made available by the authors, without undue reservation.
